# Moss Biomonitoring of Atmospheric Pollution with Trace Elements in the Moscow Region, Russia

**DOI:** 10.3390/toxics10020066

**Published:** 2022-02-02

**Authors:** Konstantin Vergel, Inga Zinicovscaia, Nikita Yushin, Omari Chaligava, Pavel Nekhoroshkov, Dmitrii Grozdov

**Affiliations:** 1 Department of Nuclear Physics, Joint Institute for Nuclear Research, Joliot-Curie 6, 141980 Dubna, Russia; verkn@mail.ru (K.V.); ynik_62@mail.ru (N.Y.); omar.chaligava@ens.tsu.edu.ge (O.C.); p.nekhoroshkov@gmail.com (P.N.); dsgrozdov@rambler.ru (D.G.); 2 Department of Nuclear Physics, Horia Hulubei National Institute for R&D in Physics and Nuclear Engineering, 30 Reactorului Str., MG-6 Bucharest-Magurele, Romania; 3 Faculty of Exact and Natural Science, Georgian Technical University, 77 Merab Kostava Street, 0171 Tbilisi, Georgia

**Keywords:** air, moss biomonitoring, Moscow region, pollution, COVID-19

## Abstract

For the first time, moss biomonitoring covering the territory of the entire Moscow region, without including Moscow, was performed in 2020. Moss *Pleurozium schreberi* collected at 156 sampling sites were analyzed using neutron activation analysis and atomic absorption spectrometry. A total of 34 elements were determined in moss samples. Obtained data were compared with the results of the moss surveys performed in the Vladimir and Yaroslavl regions in 2018 and with the data of moss surveys conducted in the Moscow region on a limited number of sampling sites in 2004 and 2014. The Moscow region showed to be more polluted than the Vladimir and Yaroslavl regions. In the the Moscow region, the decrease of the content of the main part of the elements over time was noted. Trace elements emission sources were identified and characterized using factor analysis. Contamination Factor, Pollution Load Index, and Ecological Risk were calculated to assess the level of the region contamination and elements effect on human health. In general, the Moscow region can be characterized as unpolluted to moderately polluted with a low ecological risk of human exposure. The cities satellites around Moscow were determined to experience particular environmental stress, even in the period of the COVID-19 restrictions.

## 1. Introduction

Environment pollution with heavy metals is a pressing problem for many countries in the world and its solution is addressed by different national and international organizations. To monitor and reduce air pollution, the Convention on Long-range Transboundary Air Pollution (LRTAP) was signed in 1979, which aimed to study the effects of acid rain, ozone, persistent organic pollutants, and heavy metals on air quality [1]. Within the LRTAP convention, the European Moss Survey, based on the basic research conducted by Rühling and Tyler, using naturally growing mosses as biomonitors of atmospheric deposition of trace elements has been conducted since 1990 every five years [1,2].

The application of mosses as bioindicators is explained by their widespread occurrence, morphological and physiological properties, ability to withstand adverse environmental conditions, and high sensitivity to toxicants. Mosses accumulate trace elements from the atmosphere, retaining and storing them throughout life. Since mosses do not have a well-developed root system, the contribution of sources other than atmospheric deposition in most cases is limited. Appling different analytical techniques, it is possible to determine the elemental composition of atmospheric deposition at the sampling site and to quantify the concentration of a given pollutant accumulated by moss over a certain period of time. Although the moss technique does not provide direct quantitative measurement of deposition, this information can be obtained by applying different mathematical approaches [2]. The use of mosses as indicators of atmospheric pollution has significant advantages over traditional methods since the collection of samples is simple, does not require expensive equipment for air and precipitation sampling, and the process of moss collecting, transporting, and storing is less laborious.

In the Moscow region, the monitoring of heavy metals by moss sampling and analysis started in 2009, but only at a small number of sites [3]. Transport, industrial activity, and thermal power plants were identified as main air pollution sources. In 2014, a moss survey covering 39 sites mainly in the north-eastern part of the region was carried out [4]. The studied zone was characterized as unpolluted to severely polluted with the highest level of metal content in the cities located in immediate proximity to Moscow. Since two analytical techniques (neutron activation analysis and atomic absorption spectrometry) were applied for moss samples analysis, in both surveys it was possible to determine more chemical elements than those reported in the European moss survey atlas.

In 2020, for the first time, a campaign covering the entire territory of the Moscow region was conducted with the aim to (i) determine the current content of elements in moss samples, (ii) to compare the recent deposition of trace metals with the levels recorded in the past surveys in Moscow region and other regions of the Russian Federation, (iii) to identify main air pollution sources, and (iv) to evaluate the level of air pollution using several pollution indices.

## 2. Materials and Methods

### 2.1. Studied Area

The Moscow region, located in the center of the European part of Russia, includes two constituent entities: the Moscow region and the city of Moscow. It covers an area of 44.3 thousand km^2^ with a population of 7.7 million people. In the Moscow region, there are 73 cities and 67 urban-type settlements. The studied region is located in the central part of the East European Plain, mainly in the interfluve area of the Volga and Oka rivers. The climate is moderately continental with the average monthly temperature in January −5.3 °C and in July 17.7 °C. The average annual precipitation is 480–700 mm. The main rivers are Oka, Volga, and Klyazma.

The Moscow region is one of the largest industrial regions in the country. Economically, the Moscow region is closely connected with Moscow. The main processing industries of the Moscow Region are food production, production of coke and non-oil products, chemical production, production of rubber and plastic products, production of machinery, vehicles and equipment, metallurgical production, and production of electrical and military equipment. The main industrial centers are: Electrostal, Lyubertsy, Krasnogorsk, Mytishchi, Orekhovo-Zuevo, Pavlovsky Posad, Voskresensk, Yegoryevsk, Kolomna, Podolsk, Klin, Serpukhov, Noginsk, Sergiev Posad, Dmitrov, etc. A more detailed description of the region can be found in our previous study [3].

### 2.2. Sampling and Chemical Analysis

**Figure 1:**
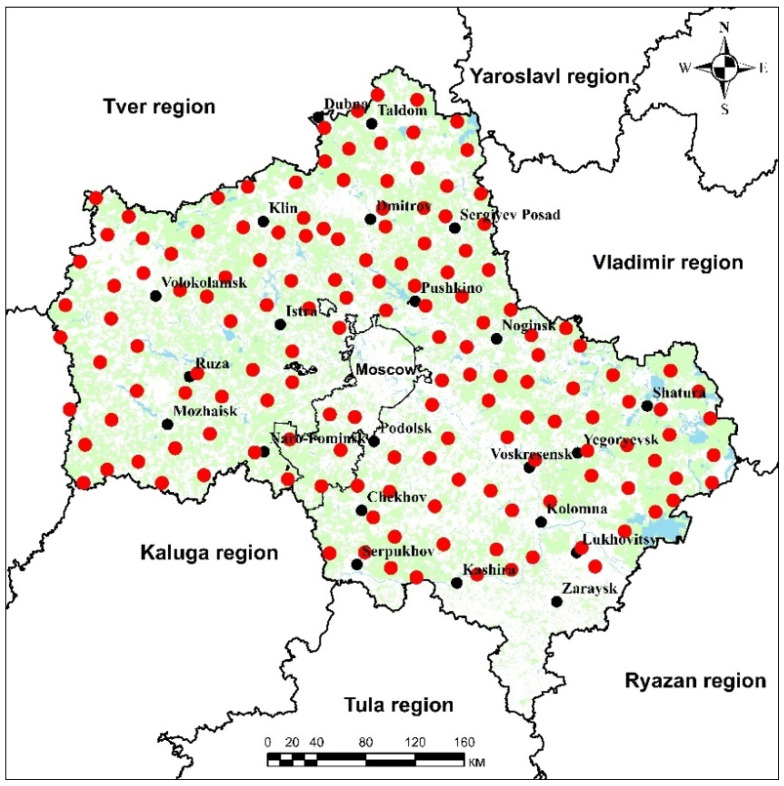
Location of the sampling sites in the Moscow region.

For monitoring of atmospheric deposition, samples of the moss *Pleurozium schreberi* were collected in the period June–August 2020 at 156 sites (the description of the sites is given in Table S1) evenly distributed across the Moscow region (Figure 1). Moss sampling was performed following the Monitoring manual “Heavy metals, nitrogen and POPs in European mosses: 2015 survey” [5]. According to the manual, each country should aim to collect at least 1.5 moss samples/1000 km^2^. In the present study, moss samples were collected in a grid with a spacing of approximately 30 km × 30 km (3.5 moss samples/1000 km^2^). Mosses were collected on the ground or surface of decaying stumps at least 3 m away from the nearest projected tree canopy. Samples were collected at a distance of least 300 m away from villages and industries, and at least 100 m from smaller roads. The main criteria regarding the sampling were: about 1.5 kg of fresh moss was collected at each sampling point, consisting of five to ten sub-samples of the same moss species. A separate set of polyethylene gloves was used for the collection of each sample. Collected samples were stored in air-permeable bags.

For elemental analyses, the collected samples were cleaned of foreign material and dried at 105 °C after preparation using the last 3 years’ growth for determining trace elements. Since 2004 neutron activation analysis (NAA) was used for the determination of the main part of elements in moss samples, in 2014, atomic absorption spectrometry (AAS) was introduced to complement the data with Cd, Pb, and Cu content. For NAA, moss samples of about 0.25 g were pelletized and packed in polyethylene foil bags for determination of elements with short-lived isotopes and in aluminum cups for determination of elements with long-lived isotopes. For AAS, samples were digested into a microwave digestion system (Mars; CEM, Waltham, MA, USA).

NAA was performed at the radioanalytical complex REGATA of the IBR-2 reactor (Dubna, Russia). Elements, Mg, Al, Cl, V, Ti, Ca, I, and Mn were determined after samples irradiation for 3 min at a neutron flux of 1.1 × 10^12^ n cm^–2^ s^–1^ and measured for 15 min. To determine Na, K, Sc, Cr, Fe, Co, Ni, Zn, As, Br, Se, Rb, Sr, Sb, Cs, Ba, La, Ce, Sm, Tb, Hf, Ta, W, Th, and U, samples were irradiated for 4 days at a neutron flux 1.1 × 10^11^ n cm^–2^ s^–1^, re-packed, and measured twice using HP-Ge detectors after 4 and 20 days of decay, respectively. Gamma spectra processing and determination of element mass fractions were performed using Genie 2000 and software developed in FLNP JINR.

The content of Cd, Cu, and Pb in the moss samples was determined by using iCE 3400 AAS Atomic Absorption Spectrometer with electrothermal (graphite furnace) atomization (Thermo Fisher Scientific, Waltham, MA, USA) [6].

A set of certified reference materials (SRM): NIST SRM 1575a (Trace Elements in Pine Needles), NIST SRM 2709 (San Joaquin Soil), NIST SRM 2711 (Montana Soil), NIST 1632c (Trace Elements in Coal (Bituminous)), CTA-FFA-1 (Fine Fly Ash), and IC-INCT-OBTL-5 (Ori-ental Basma tobacco leaves) was used for quality control. A comparison of the determined and certified values is presented in Table 1.

### 2.3. Data Evaluation

Basic descriptive statistical measures (minimum, maximum, median (MD), mean, standard deviation, 25th percentile (Q_1_), 75th percentile (Q_3_), coefficient of variation (CV), and Percentile 90) for the concentrations of determined elements in moss were calculated in Excel software. To investigate whether significant differences between the data from different monitoring campaigns exist, due to non-normal data distribution, Wilcoxon signed-rank test was applied. Factor analysis was used to establish the relationships among determined elements and to identify possible sources of their emissions.

### 2.4. Pollution Indices

The contamination factor (CF) is defined as the ratio between the content of an element in the sample and its background value [6]:

(1) $$\mathrm{CF}=\frac{C_{m}}{C_{b}}$$

where C_m_ is the content of a selected element and C_b_ is the background concentration for the same element.

Contamination degrees can be categorized as following: CF < 1—no contamination; 1–2—suspected; 2–3.5—slight; 3.5–8—moderate; 8–27—severe; and >27—extreme [6].

The PLI represents the n^th^ order geometric mean of the entire set of CF values [7]:

(2) $$\;\mathrm{PLI}=\sqrt[{n}]{\prod_{i=1}^{n}C_{F,i}\;}$$

where n is the total number of elements.

The PLI data were classified in several groups: PLI < 1—unpolluted, 1 < PLI < 2—unpolluted to moderately polluted, 2 < PLI < 3—moderately polluted, 3 < PLI < 4—moderately to highly polluted, 4 < PLI < 5—highly polluted, and PLI > 5—very highly polluted [7].

Ecological risk index (RI) is used to measure the ecological risk of a given element in moss according to the toxicity of metals and response of the environment:

(3) $$\mathrm{RI}=\sum {\mathrm{PER}}_{f}^{i}$$

(4) $${\mathrm{PER}}_{f}^{i}={\;C}_{f}^{i}\times {\;T}_{f}^{i}$$

where ${\mathrm{PER}}_{f}^{i}\;$ is the potential ecological risk index of each element, $C_{f}^{i}\;$ is the contamination factor, and $T_{f}^{i}$ is the “toxic-response” coefficient for the given single metal. The toxic response factors are 2 for Cr, 6 for Ni, 5 for Cu, 10 for As, 30 for Cd, 1 for Zn, and 5 for Pb [8]. The ecological risk according to its severity was classified into four groups: RI < 150—low ecological risk; 150 ≤ RI < 300—moderate ecological risk; 300 ≤ RI < 600—considerable ecological risk; RI ≥ 600—very high ecological risk [8].

## 3. Results and Discussion

The results of descriptive statistics for 34 chemical elements determined in moss samples are presented in Table 2. The coefficient of variation values calculated for all the determined elements varied from 24.7% to 78.2%, indicating a moderate variation. The highest variabilities of 78.2%, 64.7%, and 63.1% were obtained for W, Sb, and Cl, respectively. It is considered that a moderate variation reflects similar contamination levels for all elements throughout the studied region and indicates the stability of their content in mosses [9].

The comparison of the results obtained in the present study with the results of the previous moss surveys performed in 2004 and 2014 in the Moscow region, as well as in Vladimir and Yaroslavl regions in 2018, is given in Table 3.

The median values of the main part of the elements, except As, Br, Rb, and Se, in the Moscow region in 2020 were approximately 10–50% higher than in the Yaroslavl region. The highest differences were observed for Al (44.4%), Hf (46.1%), Zn (40.3%), Pb (41.9%), and U (44.2%). The same pattern was observed for the Vladimir region, where median values of all elements, except Mg and Cd, were lower than in the Moscow region (2020). The most significant differences were obtained for Ca (53%) and Sc (64%). It should be mentioned that mean values for V, Cr, Mn, and Ni obtained for both regions were comparable (difference not more than 4%). Higher median values obtained for the Moscow region are explained by high traffic density and the operation of a large number of production enterprises.

In comparison with the moss campaign performed in the Moscow region in 2004, lower median values for almost all elements, except Al, Ca, Sc, Mn, Zn, and Sb, were obtained in the campaign conducted in 2020. The difference in elements content between the two surveys was at the level of 9–114%. Similar patterns have been observed in between moss surveys performed in 2014 and 2020. The median values for the greater part of elements in 2020, except Mn, Cu, Zn, Se, Pb, and Br, were lower than in 2014. The difference varied from 3.9% for Mg to 161% for W. Lower values for the greater part of elements obtained in the moss survey conducted in 2020 can be explained by a large number of samples collected on the territory of the entire region. In 2004 and 2009, moss samples were collected mainly in the northern and eastern parts of the region, where a large proportion of industrial companies are operated. The decrease of the content of a part of elements in 2020 can be also associated with the restriction adopted by the Government of the Moscow region in order to prevent the spread of COVID-19. Thus, in the period March–June 2020, a strict self-isolation regime was introduced when only essential services and industrial enterprises did not cease to operate; however, the number of vehicles has dropped significantly.

In the moss survey conducted in 2020, 39 sampling sites coincided with sampling sites from the moss survey performed in 2014. Wilcoxon test was used to reveal differences between values obtained in two surveys. The insignificant differences (*p* > 0.05) in K, Ca, Zn, Se, Rb, Sb, Ba, and Cu content for selected collection sites were observed, and the values of other elements were significantly different (*p* < 0.05).

### 3.1. Factor Analysis

**Figure 2:**
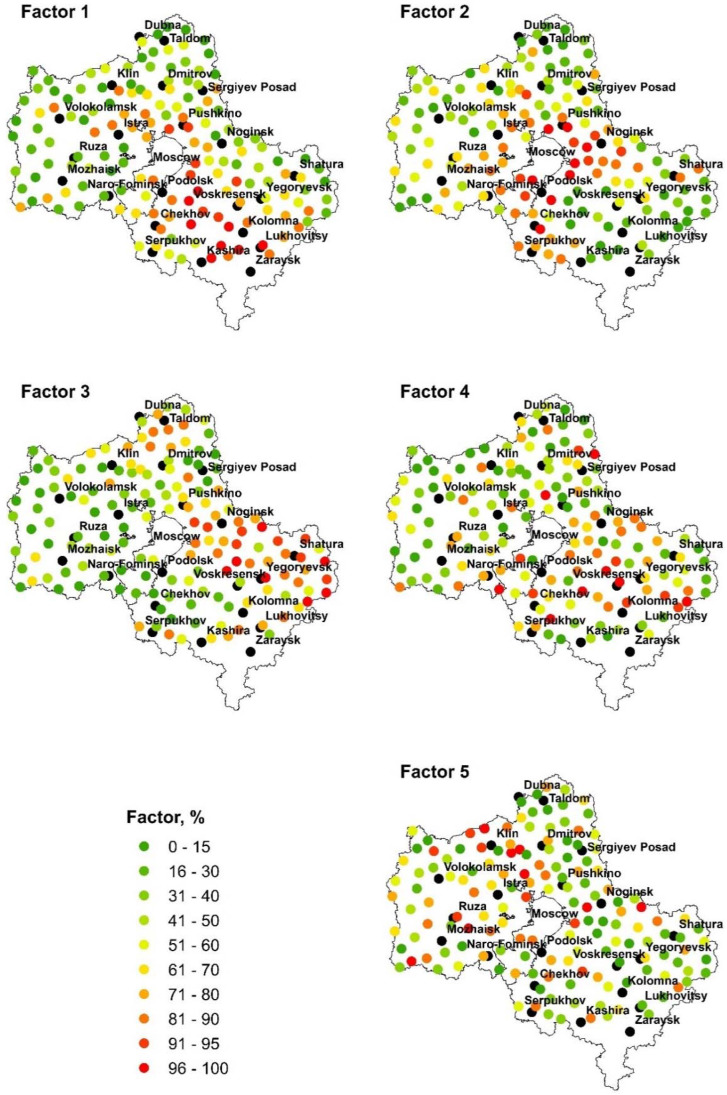
Spatial distribution of Factors 1–5 scores.

Factor analysis with varimax raw rotation was applied to identify elements associations and to connect them with possible sources of pollution. Five factors were extracted, including 80% of the variability of the treated elements. The matrix of rotated factor loadings is given in Table 4 and graphically presented in Figure 2.

Factor 1 (F1) represents 43% of the total variance and can be defined as a geogenic and anthropogenic association of elements (Figure 2). The highest concentrations of elements contributing to F1 (Na, Al, Sc, V, Cr, Fe, Co, Ni, As, La, Ce, Sm, Tb, Hf, Ta, Th, and U) were determined in moss samples collected in the south-west parts of the region. In order to distinguish the lithogenic and anthropogenic origin of the elements in, F1 scandium was used as a normalizing trace element. Scandium is a typical widespread trace element in the earth’s crust [11].

**Figure 3:**
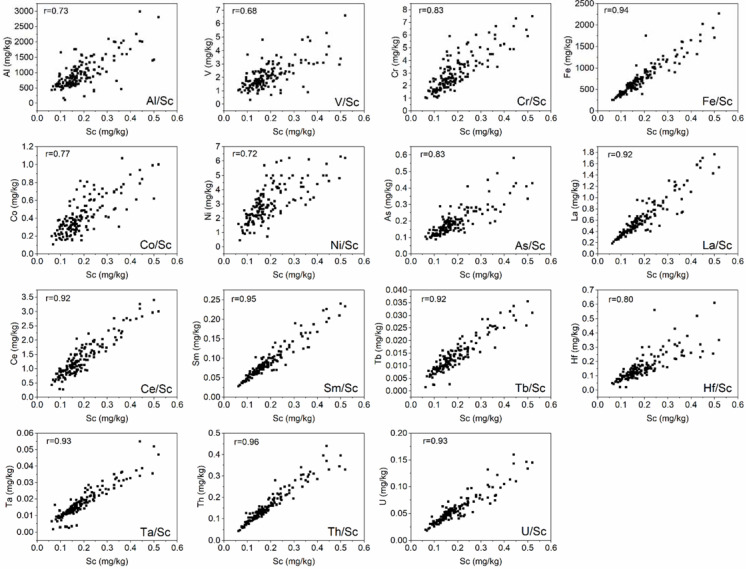
The ratio of the content of each element in Factor 1 versus Sc content in moss samples.

High values of the Pearson coefficient (0.8–0.92) obtained for Na/Sc, Fe/Sc, La/Sc, Ce/Sc, Sm/Sc, Tb/Sc, Ta/Sc, U/Sc, and Th/Sc ratios (Figure 3) indicated the geogenic origin of these elements. The weak correlation obtained for V/Sc, Al/Sc, Co/Sc, Ni/Sc, As/Sc, and Hf/Sc ratios may be explained by their anthropogenic origin. The association of V, Ni, Al, and As may originate from the combustion of coal, diesel oil, fuel oil, and the incineration of waste and sewage [12]. Hafnium is an element widely used in the nuclear industry and space power systems. Hafnium is also used for alloying with iron, titanium, aluminum, and other metals [13,14]. The possible sources of Hf emissions are located in Korolev, Reutov, and other cities located near Moscow.

Vanadium and nickel are important tracers of oil combustion. The values of the V/Ni ratio in 75% of the sampling sites in the Moscow region lie in the range of 0.7–1.1. In 20% of the sites, they varied from 0.14 to 0.4, indicating the presence of a specifically Ni-rich atmospheric pollution source [15]. This assumption is confirmed by Ni with almost the same factor loading in Factor 5. The candidates for such a source are metallurgical and engineering plants located in Stupino, Voskresensk, etc. In 5% of the sampling sites, which were close to refinery companies, for example, Orekhovo-Zuyevo, the V/Ni ratio varied from 2.1 to 2.9. This is in agreement with Pacyna and Lindgren’s study [16].

The second factor (F2) represented 13% of the total variance and was associated with high loads of Cr, Fe, Co, Ni, Sb, and W. The highest concentration of mentioned elements was determined in the city’s satellites around Moscow, where the main part of industrial enterprises is located. Thus, this association of elements can derive from metallurgical plants, machinery companies, plants for nuclear fuel production, and chemical and pharmaceutical enterprises located in Staraya Kupavna, Mytischi, Balashikha, Zheleznodorozhniy, Electrostal, Podolsk, Odintsovo, etc. The high traffic density in this zone is another important contributor to heavy metals emission.

Obtained results are in agreement with the data obtained in the previous moss campaign [3]. The coefficients of the correlation of Cr/Sc, Co/Sc, Sb/Sc, W/Sc, Ni/Sc, and Al/Sc ratios (Figure S1) confirmed their anthropogenic nature.

The third factor includes Rb and Cs and represented 8% of the total variance. The correlation coefficients obtained for the ratios Rb/Sc and Cs/Sc were extremely low (0.02–0.04), indicating their anthropogenic origin (Figure S2). The highest concentrations of mentioned elements were determined in the eastern part of the Moscow region (Mytischi, Balashikha, Zheleznodorozhniy, Electrostal, Vosrkesensk, Yegoryevsk, etc.), where a great number of industrial enterprises are operated. Both elements are widely applied in fiber optics, telecommunication systems, and night-vision devices also for glass and electronic device production [17,18,19]. Rubidium is also applied in the pharmaceutical industry and medicine to produce sleeping pills and sedatives and in the treatment of bipolar disorders [17,18]. One of the pharmaceutical companies, “Endo-pharm -a- PP”, is located in Shchelkovo. Around seven glass-producing companies, including “Glass technologies” in Electrostal and ”Glass and glass articles” in Lyubertsy, are located in the eastern part of the region.

Factor 4 represents 8% of the total variance and is associated with high loads of Zn, Cu, Pb, and Cd (Figure 2). Since the coefficients of correlation of Zn/Sc, Cu/Sc, Pb/Sc, and Cd/Sc ratios were very low (Figure S3), this association of elements can be explained by the high-density traffic in densely populated areas [20]. According to [21], Pb may be originally from yellow and red road markings, and gray paint or anticorrosives. Lead is an important component of bearing alloys [22]. Even the use of leaded gasoline in Russia was prohibited for use in 2002, and its relatively high concentrations can be explained by lead persistence in the environment. Cadmium and Zn are mainly emitted from the abrasion of tire rubber, while diesel soot is another important source of Zn [21]. Copper contamination could originate from the frictional materials used in the brake system [22].

Despite the fact that during the period of self-isolation traffic in the Moscow region declined by 50% (according to the reports by the national authorities), transport remains one of the major sources of air pollution in the region.

Factor 5 (Figure 2) represented 8% of the total variance and was loaded by Mg, Al, and Ca. The main source of these elements can be considered dust from soil erosion. However, since the values of the coefficient of correlation obtained for Ca/Sc, Mg/Sc, and Al/Sc were low (0.11–0.56) (Figure S4), it can be assumed that contributors of these elements are industrial sources, mining, and fuel combustion. Today, approximately 140 deposits of solid minerals (sand, dolomites, grit, clays, limestones, phosphates, potassium salt, etc.) are developed on the territory of the Moscow region. On the territory of the Ruza district, where high concentrations of Ca, Mg, and Al were determined, there are up to 60% of the mineral resources of the Moscow region. Seventeen deposits are developed in the Solnechnogorsk district, 25 in Mozhaisk, and 24 in the Klin district. Road dust is another important source of Ca, Al and Mg, along with sulfates, chlorides, nitrogen compounds, phosphates, K, Na, and heavy metals [23]. Ca/Al ratio can be taken as a tracer to distinguish the geological sources from urban or non-urban ones. In the present study, the values of the Ca/Al ratio varied from 1.6 to 14, indicating influence from anthropogenic emissions, for example, construction activities.

Aluminum, Cr, Fe, Co, and Ni were elements characterized by high factor loadings in several factors, which may indicate multiple sources of their atmospheric emissions.

### 3.2. Pollution Indices

To evaluate the level of environmental pollution, the contamination factor (CF) and PLI (Pollution Load Index) were calculated. Both indices were calculated for elements which, according to rations of their content versus Sc content, had low values of coefficients of determination and are considered to be emitted as the result of anthropogenic activity (Table 5).

Based on the mean CF values (Table 4), the Moscow region can be characterized by two categories of contamination scales, described as no contamination and suspected contamination, respectively. However, at some sampling sites, the values of CF varied from 3.5 to 8 and pointed at moderate contamination. Thus, the high CF for W (4.7–8), Sb (3.5–7.1), Fe (3.3–3.9), and As (3.3–3.9) were obtained near Balashiha, Staraya Kupavna, Kolomna, and Lyubertsy. The highest values of CF for Cu (7.0) and Cd (4.4), indicating moderate contamination, were obtained in Sergiyev Posad, where electromechanical and optical mechanics plants, and plastic and chemical production companies are located. According to PLI values (Table 4), the Moscow region can be characterized as unpolluted to moderately polluted.

The potential ecological risk index (PER) and risk index (RI) were calculated to assess the ecological hazards in the Moscow region. The PER values for Cr (2.6 ± 1.2), Cu (6.7 ± 3.1), Zn (1.5 ± 0.6), As (15.9 ± 6.4), and Pb (8.2 ± 4.2) were lower than 40, indicating low potential ecological risk, and the values obtained for Cd (47.5 ± 17) pointed at moderate potential ecological risk. RI values ranged from 33 to 164, with an average of (82 ± 25) indicating low or moderate ecological risk. The highest RI values were obtained for Vorkresensk, Kolomna, Stupino, and Domodedovo cities, indicating negative effects of the industrial activities on the air quality. In Voskresensk, Stupino, and Domodedovo, there are large industrial enterprises such as metallurgical and engineering plants, chemical factories, electromechanical and mechanical plants, paint and coatings production plants, etc. The main parts of the enterprises operated even in the period of the COVID-19 restrictions, contributing to pollutant emission in the atmosphere.

## 4. Conclusions

The third study of atmospheric deposition of trace elements in the Moscow region and the first covering the territory of the whole region, without including Moscow, using moss biomonitoring technique, was performed in 2020. The content of 35 chemical elements was determined in moss samples using neutron activation analysis and atomic absorption spectrometry. Comparison of the results obtained for the limited number of samples collected in the Moscow region in 2014 and 2020 revealed a significant decrease in the content of the main part of elements in 2020, indicating the improvement of the environmental situation mainly in the northeast part of the region. The Moscow region showed to be more polluted than the Vladimir and Yaroslavl regions. Factor analysis allowed extraction of five factors: F1 characterized as geogenic-anthropogenic associations of elements and F2–F5—anthropogenic factors. The main sources of air pollution in the Moscow region can be considered industrial activity, transport, mining, and construction. According to CF and PLI values, the environmental status of the region may be characterized as unpolluted or moderately polluted. The important contaminants of air in the Moscow region are city satellites around Moscow, where a large number of industrial enterprises of different assignments operate. The measures adopted to decrease the spread of COVID-19 resulted in the reduction of the level of air pollution in the northeast part of the Moscow region; however, they did not affect emissions in the city satellites around Moscow, where the main parts of enterprises continued operating.

## Figures and Tables

**Table 1:** Quality control of neutron activation analysis and atomic absorption spectrometry.

Element	SRMs	Concentrations, ppm		Uncertainties, %	
		Determined	Certified	Determined	Certified
Na	2709a	11,934	12,200	7.8	2.5
Mg	1575a	1345	1060	7.6	16
Al	1632c	8896	9150	6.3	1.5
Cl	1575a	417	421	3.4	1.7
K	1632c	1590	1100	2.3	3
Ca	1575a	2427	2500	8.2	4
Sc	1632c	2.92	2.91	3.2	1.2
V	1632c	17.3	23.7	5.9	2.2
Cr	1632c	15.23	13.73	8.5	1.5
Mn	1632c	13.8	13.04	9.4	4
Fe	2709a	32,595	33,600	5.5	2.1
Co	2709a	12.3	12.8	5.3	1.6
Ni	2709a	10.8	9.32	8.8	5.5
Zn	1632c	11.7	12.1	9.4	10.7
As	FFA1	53.4	53.6	5.4	5
Se	1632c	1.3	1.3	43	5.4
Br	1632c	20.8	18.7	4.4	2.1
Rb	2709a	96.6	99	6.6	3
Sr	1632c	63.1	63.8	7.7	2.2
Sb	FFA1	17.9	17.6	6.1	14.2
Cs	FFA1	46.5	48.2	3.7	5.4
Ba	1632c	43.5	41.1	4.4	3.9
La	2709a	20.9	21.7	7.4	1.8
Ce	1632c	12.2	11.9	7.2	1.7
Sm	FFA1	10.2	10.9	9.1	5.5
Tb	FFA1	1.365	1.38	4.1	10.1
Hf	FFA1	7.75	6.09	5.5	7.4
Ta	FFA1	1.83	2.11	3.2	7.6
W	FFA1	10.5	10.5	11.1	10.5
Th	2709a	9.97	10.9	4.8	1.8
U	FFA1	13.6	15.1	3.9	5.3
Cd	IC-INCT-OBTL-5	2.5	2.64	5.5	5.3
Pb	IC-INCT-OBTL-5	1.94	2.01	7.2	15.4
Cu	IC-INCT-OBTL-5	9.8	10.1	4.3	4.0

**Table 2:** Descriptive values for major and trace elements in mosses collected in 2020 in the Moscow region, in mg/kg.

Element	Range	Median	Mean ± st.dev	Q1	Q3	CV (%)	Percentile 90
Na	85–508	155	177 ± 76	126	202	43.0	296.6
Mg	166–2970	1790	1762 ± 498	1460	2070	28.3	2418
Al	108–2990	853	993 ± 487	656	1190	49.1	1648
Cl	9.6–284	85.0	87 ± 55	54.0	112	63.1	146
K	493–14,300	7230	7388 ± 1824	6110	8390	24.7	9690
Ca	727–9050	4480	4611 ± 1490	3610	5670	32.3	6610
Sc	0.06–0.52	0.17	0.20 ± 0.09	0.14	0.23	47.8	0.34
V	0.32–5.3	1.90	2.0 ± 0.9	1.44	2.50	44.1	3.28
Cr	1.01–7.5	2.63	3.1 ± 1.4	2.10	3.80	45.7	5.17
Mn	0.46–1540	449	462 ± 258	293	577	55.9	748.6
Fe	254–2270	690	784 ± 380	531	941	48.5	1304
Co	0.11–1.07	0.38	0.4 ± 0.2	0.28	0.52	47.2	0.72
Ni	0.46–6.3	2.87	3.05 ± 1.28	2.20	3.90	42.1	5.00
Zn	1.3–145	57.0	62.5 ± 23.4	47.0	75.0	37.5	89.6
As	0.03–0.49	0.18	0.20 ± 0.08	0.14	0.23	40.6	0.29
Se	0.04–0.36	0.17	0.17 ± 0.05	0.14	0.20	31.9	0.24
Br	1.07–4.4	2.26	2.37 ± 0.66	1.90	2.80	28.0	3.40
Rb	0.14–39.5	13.8	16.48 ± 9.5	8.60	23.5	57.7	31.9
Sr	4.2–30.5	15.3	15.8 ± 5.5	12.3	19.4	35.0	23.0
Sb	0.0048–1.13	0.23	0.27 ± 0.18	0.16	0.34	64.7	0.50
Cs	0.0062–0.47	0.14	0.16 ± 0.08	0.097	0.20	49.3	0.28
Ba	3.1–113	44.0	46.1 ± 22.1	29.0	59.0	47.9	79.8
La	0.19–1.76	0.54	0.63 ± 0.32	0.41	0.73	51.1	1.12
Ce	0.27–3.4	1.20	1.37 ± 0.63	0.91	1.70	46.0	2.29
Sm	0.028–0.24	0.08	0.09 ± 0.04	0.064	0.11	47.2	0.16
Tb	0.0015–0.04	0.013	0.014 ± 0.007	0.0096	0.016	47.1	0.025
Hf	0.02–0.61	0.13	0.16 ± 0.09	0.10	0.19	58.9	0.28
Ta	0.0018–0.06	0.016	0.018 ± 0.0095	0.012	0.022	52.9	0.032
W	0.04–1.13	0.18	0.22 ± 0.18	0.12	0.24	78.2	0.44
Th	0.04–0.44	0.14	0.16 ± 0.08	0.11	0.19	48.0	0.29
U	0.0029–0.16	0.052	0.057 ± 0.027	0.039	0.066	47.3	0.097
Cd	0.08–0.54	0.24	0.25 ± 0.09	0.18	0.31	37.0	0.39
Pb	1.33–14	4.82	5.28 ± 2.71	3.29	6.44	51.3	8.99
Cu	3.03–43	7.61	8.23 ± 3.81	6.26	9.41	46.3	11.6

**Table 3:** A comparison of the content of the elements as obtained in the present study and literature data. All contents expressed in mg/kg except Al, K, Ca, and Fe, whose contents are expressed in % d.w.).

Ref.	Moscow Region (Present Work)		Moscow Region (Vergel et al. 2019)		Moscow Region (Vergel et al. 2009)		Vladimir Region (Vergel et al. 2014)		Yaroslavl Region	
	156 (present study)		39 [3]		34 [4]		73 [10]		53 [10]	
Element	MD	Range	MD	Range	MD	Range	MD	Range	MD	Range
Na	155	85–508	230	71–726	240	87–1716	128	75–942	98	56–290
Mg	1790	166–2970	1860	1010–4970	1963	364–5412	1910	1020–3030	1370	880–2150
Al	0.09	0.011–0.3	0.12	0.045–0.69	0.08	0.03–0.92	0.065	0.019–0.23	0.05	0.033–0.17
Cl	85	10–284	108	47–1040	182	54–815	68	9–434	68	39–200
K	0.72	0.05–1.43	0.85	0.23–1.7	1.08	0.55–2.26	0.47	0.47–1.4	0.63	0.44–0.9
Ca	0.45	0.07–0.91	0.47	0.24–0.9	0.35	0.12–0.92	0.21	0.21–0.78	0.34	0.2–0.53
Sc	0.17	0.06–0.52	0.26	0.08–1.3	0.16	0.036–2	0.06	0.06–0.59	0.14	0.06–0.31
Ti	–	–	146	35–1050	–	–	–	–	68	20–141
V	1.9	0.32–5.3	2.5	0.94–11	2.3	0.68–13	1.9	0.95–6.3	1.7	0.8–8
Cr	2.63	1.01–7.5	3.2	0.72–9.5	3.1	0.51–22	2.5	1.3–7	1.8	0.39–5.8
Mn	449	0.46–1540	347	76–848	405	43–1222	431	118–931	382	48–964
Fe	0.07	0.025–0.23	0.1	0.03–0.34	0.08	0.02–0.57	0.05	0.025–0.16	0.047	0.023–0.11
Co	0.38	0.11–1.07	0.56	0.14–2.1	0.34	0.04–2.1	0.38	0.18–0.86	0.29	0.13–0.87
Ni	2.87	0.46–6.3	3.2	0.66–8.4	2.4	0.83–9	2.8	1.24–5.7	1.83	0.8–6.5
Cu	7.61	3.03–43	7.1	2.9–21	–	–	6.1	4.3–9.3	5.8	3.7–10
Zn	57	1.3–145	50	21–159	51	21–115	48	32–98	34	23–169
As	0.18	0.03–0.49	0.32	0.12–1.1	0.19	0.04–0.89	0.16	0.01–0.5	0.46	0.23–1.0
Se	0.17	0.04–0.36	0.16	0.09–0.4	0.18	0.005–0.6	–	–	0.19	0.08–1.1
Br	2.26	1.07–4.4	1.9	0.7–5.1	1.7	0.7–5.1	2.2	1.1–5	3.1	1.98–4.45
Rb	13.8	0.14–40	19	7.5–36	17	7.4–65	11	3.7–50	15	4.65–71
Sr	15.3	4.2–31	17	5.6–32	17	7.7–50	13	6.1–66	11	6.2–23
Mo	–	–	0.18	0.06–1.9	0.37	0.18–1	–	–	–	–
Cd	0.24	0.08–0.54	0.3	0.12–0.67	–	–	0.29	0.14–0.67	0.15	0.082–0.43
Sb	0.23	0.005–1.13	0.3	0.045–1.5	0.22	0.08–0.96	0.15	0.073–0.43	0.11	0.06–0.29
I	–	–	1.5	0.36–2.4	–	–	–	–	0.5	0.2–0.78
Cs	0.14	0.006–0.47	0.18	0.1–0.7	0.16	0.06–0.62	0.12	0.06–0.4	0.1	0.05–0.35
Ba	44	3.1–113	48	7.5–188	48	7.3–203	36	5.5–93	30	2.34–218
La	0.54	0.19–1.76	0.84	0.26–4.2	0.67	0.12–8.5	0.44	0.17–2.6	0.4	0.2–2.1
Ce	1.2	0.27–3.4	1.6	0.57–7.5	2.1	0.07–25	1.0	0.49–4.4	0.72	0.32–2.1
Sm	0.08	0.03–0.24	0.13	0.04–0.72	0.12	0.019–1.4	0.056	0.03–0.39	0.05	0.025–0.16
Tb	0.013	0.001–0.04	0.02	0.005–0.1	0.013	0.002–0.19	0.01	0.004–0.05	0.008	0.003–0.02
Hf	0.13	0.02–0.61	0.29	0.068–2.4	0.15	0.026–2.7	0.09	0.017–0.6	0.07	0.0005–0.2
W	0.18	0.04–1.13	0.47	0.11–200	0.35	0.08–0.78	0.1	0.02–0.53	–	0.04–0.28
Pb	4.82	1.33–14	0.67	0.12–2.2	–	–	4.2	1.9–8.8	2.8	0.003–0.07
Th	0.14	0.04–0.44	0.23	0.067–1.5	0.19	0.036–2.6	0.11	0.03–0.7	0.1	1.2–9.5
U	0.052	0.003–0.16	0.08	0.01–0.19	0.08	0.008–0.6	0.04	0.01–0.17	0.029	0.058–0.25

**Table 4:** Matrix of rotated factor loadings.

Element	Factor 1	Factor 2	Factor 3	Factor 4	Factor 5
Na	0.82	0.40	−0.01	0.09	0.13
Mg	0.12	0.04	−0.32	0.06	0.81
Al	0.72	0.09	0.16	0.12	0.52
Ca	0.14	0.13	−0.20	0.06	0.86
Sc	0.88	0.41	0.01	0.14	0.12
V	0.60	−0.01	0.35	0.17	0.49
Cr	0.63	0.56	0.25	0.17	0.11
Fe	0.75	0.57	0.13	0.16	0.09
Co	0.52	0.64	−0.01	0.08	0.19
Ni	0.56	0.52	−0.20	0.26	0.03
Zn	0.14	−0.05	0.09	0.74	0.14
As	0.77	0.20	0.17	0.15	−0.02
Rb	−0.18	0.12	0.82	0.03	−0.23
Sb	0.49	0.70	0.18	0.16	−0.01
Cs	0.13	0.08	0.83	0.02	−0.15
La	0.92	0.21	0.05	0.17	0.10
Ce	0.87	0.28	0.01	0.18	0.15
Sm	0.93	0.25	0.00	0.15	0.12
Tb	0.88	0.26	−0.12	0.16	0.08
Hf	0.84	0.02	−0.21	0.12	0.20
Ta	0.83	0.33	−0.10	0.12	0.07
W	0.49	0.75	0.22	0.07	0.05
Th	0.92	0.26	−0.02	0.13	0.13
U	0.88	0.32	0.04	0.12	0.07
Cd	0.45	0.05	−0.33	0.55	0.04
Pb	0.42	0.18	0.30	0.67	−0.18
Cu	0.00	0.36	−0.06	0.70	0.09
Prp.Totl	0.43	0.13	0.08	0.08	0.08

**Table 5:** Mean ± standard deviation values of Contamination factor and Pollution Load Index.

Element	Mean	SD	Element	Mean	SD
Mg	1.07	0.30	As	1.59	0.64
Al	1.47	0.72	Rb	0.97	0.56
Ca	0.97	0.31	Sb	1.90	1.23
V	1.48	0.65	Cs	0.97	0.48
Cr	1.31	0.60	W	1.58	1.23
Fe	1.34	0.65	Cd	1.58	0.58
Co	1.06	0.50	Pb	1.64	0.84
Ni	1.50	0.63	Cu	1.33	0.62
Zn	1.52	0.57	PLI	1.12	0.17

## Data Availability

Not applicable.

## Supplementary Materials

The following supporting information can be downloaded at: www.mdpi.com/article/10.3390/toxics10020066/s1, Figure S1: The ratio of the content of each element in Factor 2 versus Sc content in moss samples, Figure S2: The ratio of the content of each element in Factor 3 versus Sc content in moss samples, Figure S3: The ratio of the content of each element in Factor 4 versus Sc content in moss samples, Figure S4. The ratio of the content of each element in Factor 5 versus Sc content in moss samples; Table S1: Information about sampling sites.
