# Supplementary material for: Moss Biomonitoring of Atmospheric Pollution with Trace Elements in the Moscow Region, Russia
Source: Toxics. 2022 Feb 2;10(2):66. doi: 10.3390/toxics10020066 (PMC8875797; doi:10.3390/toxics10020066)
Supplement: Supplementary file 1 [file toxics-10-00066-s001.zip › toxics-1554165-supplementary.pdf]

toxics

MDPI

Supplementary Materials: Moss Biomonitoring of Atmospheric Pollution with Trace Elements in the Moscow Region, Russia

Konstantin Vergel, Inga Zinicovscaia, Nikita Yushin, Omari Chaligava, Pavel Nekhoroshkov and Dmitrii Grozdov

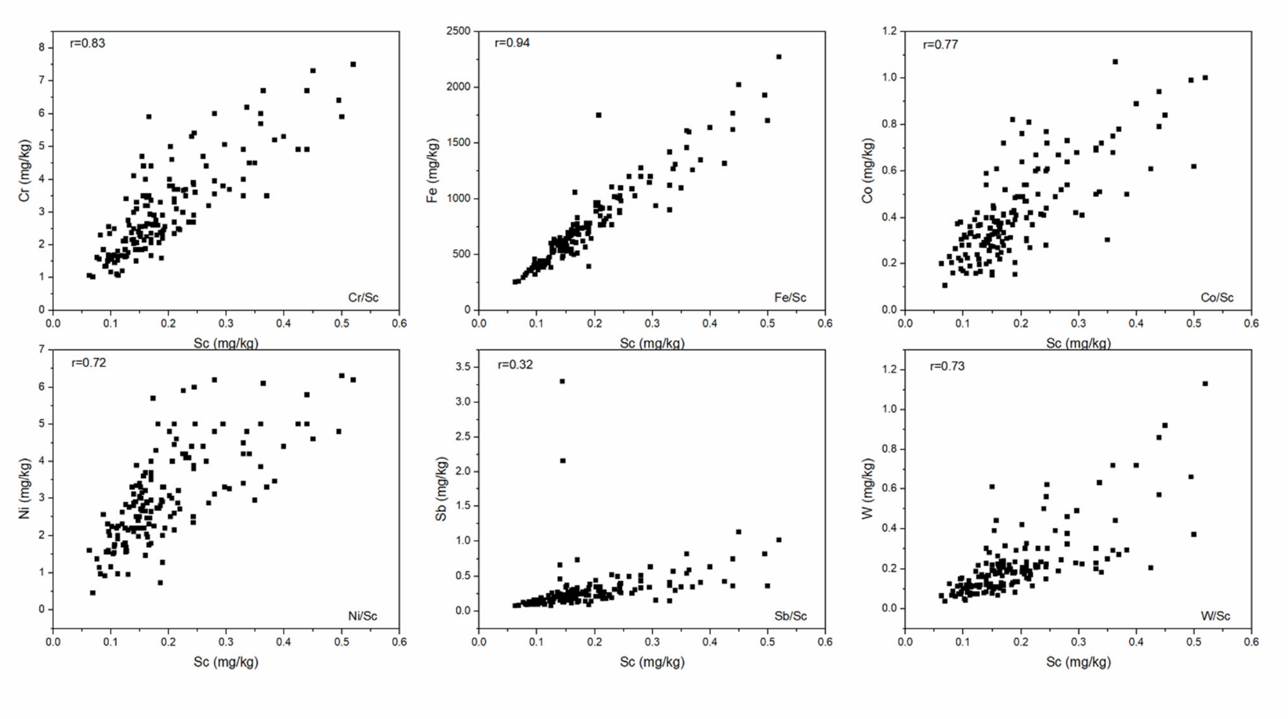

Figure S1. The ratio of the content of each element in Factor 2 versus Sc content in moss samples.

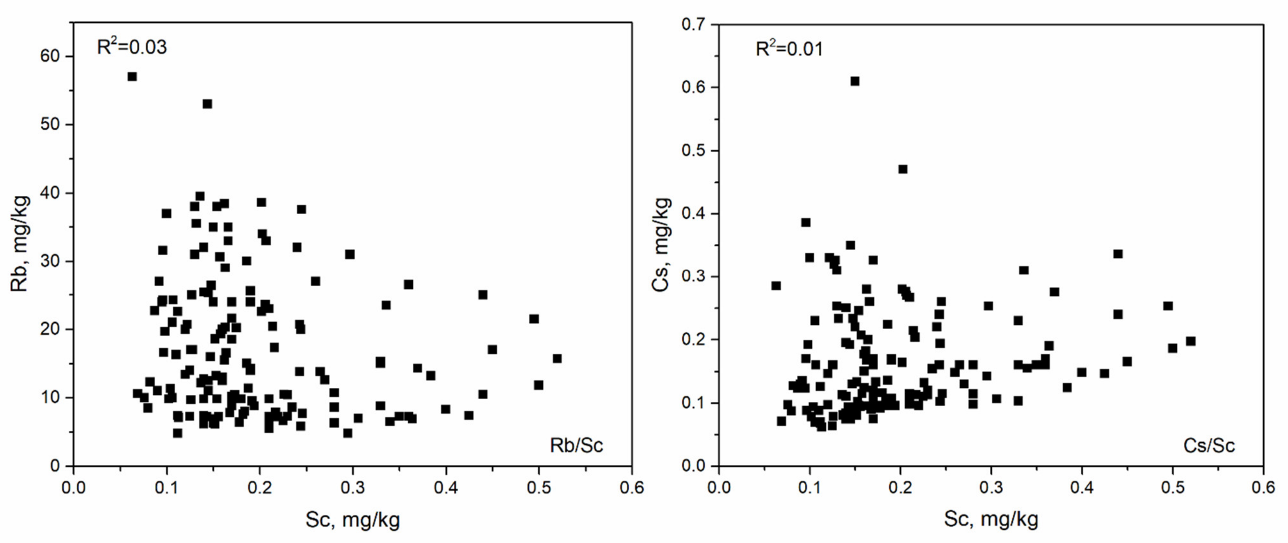

Figure S2. The ratio of the content of each element in Factor 3 versus Sc content in moss samples.

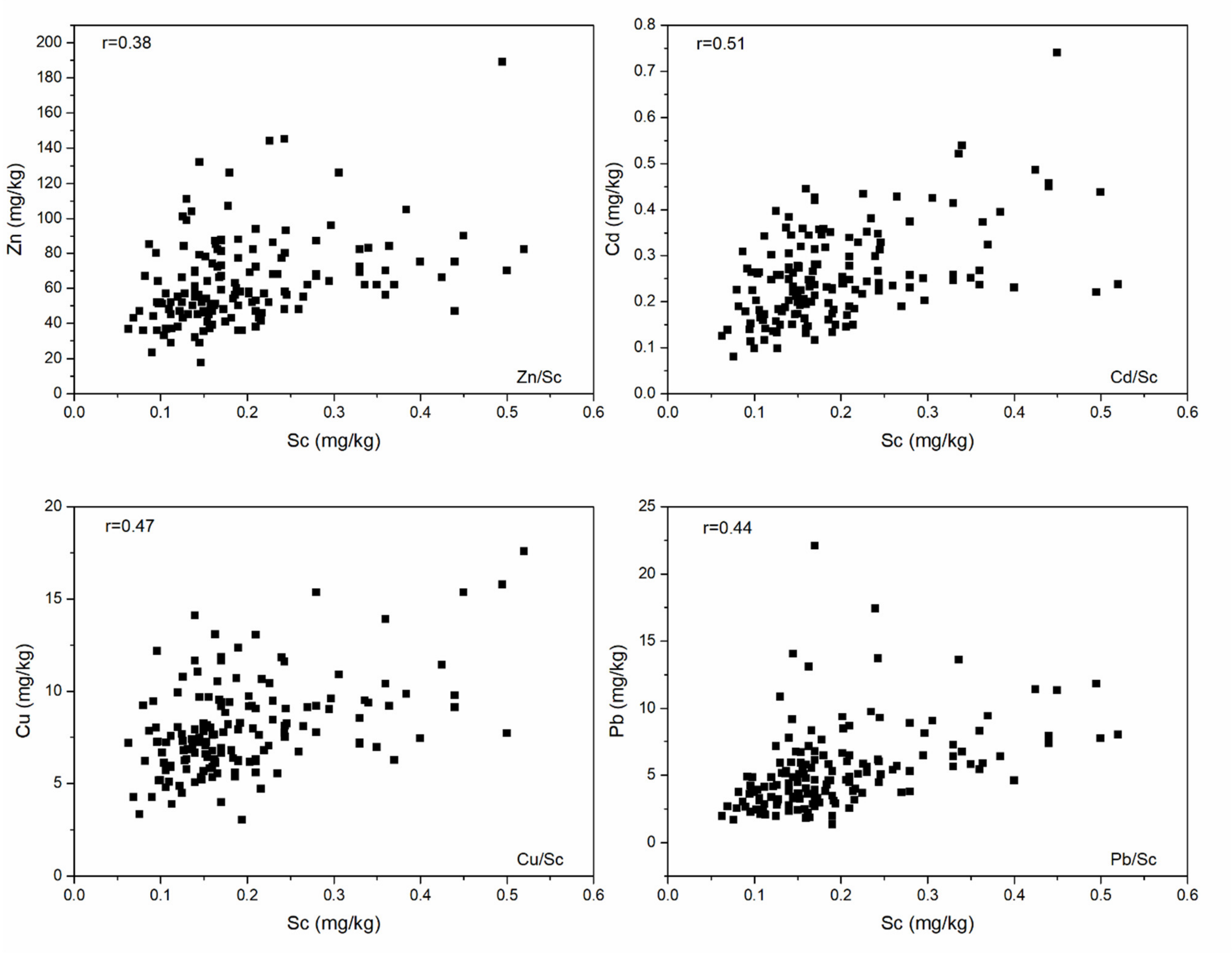

Figure S3. The ratio of the content of each element in Factor 4 versus Sc content in moss samples.

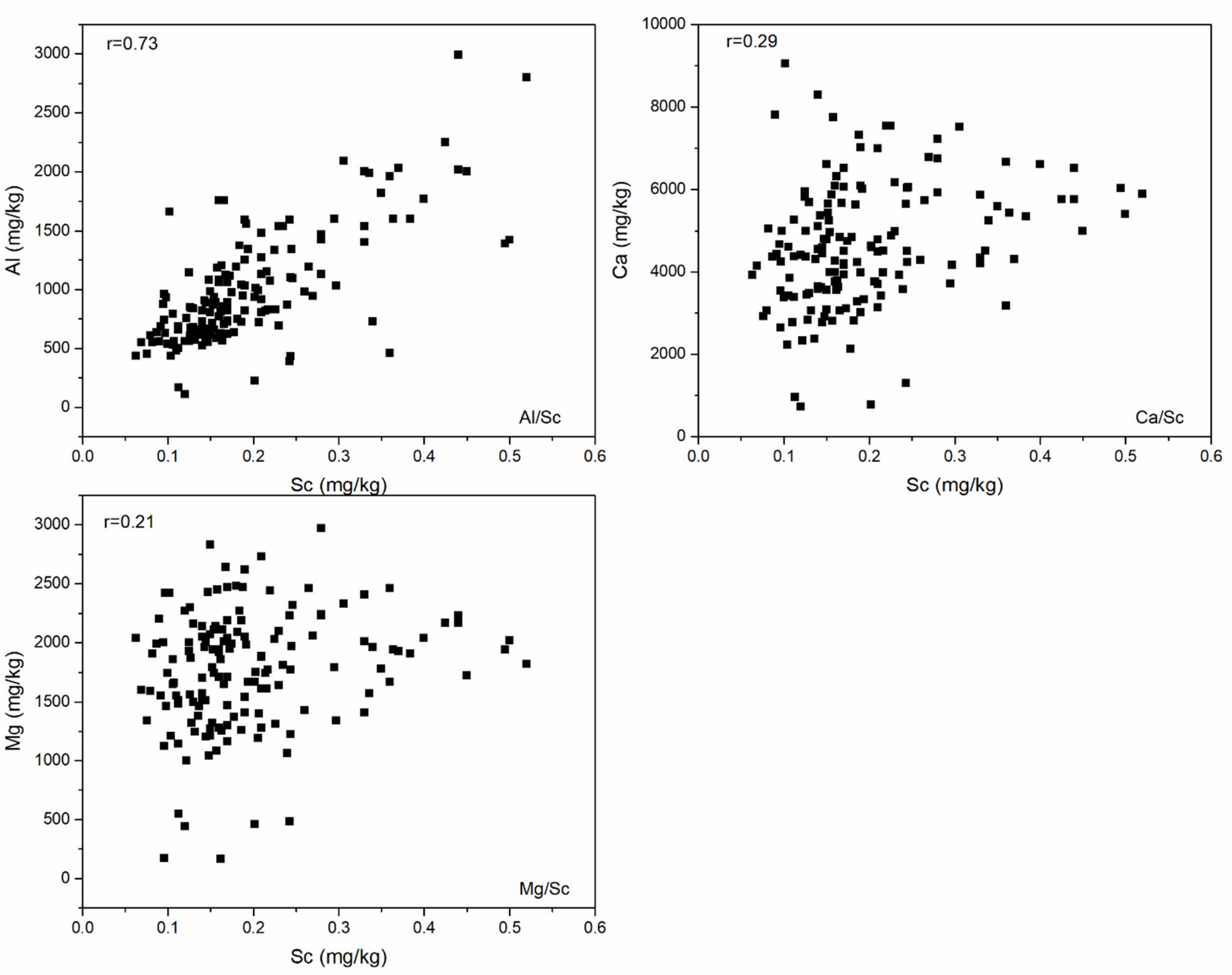

Figure S4. The ratio of the content of each element in Factor 5 versus Sc content in moss samples.

Table S1 Information about sampling sites

Sample site	Coordinates		Description
	Longitude	Latitude	
1	37,356	56,486	Coniferous forest
2	37,187	56,580	Mixed forest
3	36,974	56,495	Forest belt near agricultural field
4	36,622	56,479	Mixed forest
5	36,368	56,422	Mixed forest
6	36,588	56,319	Spruce and birch forest
7	36,709	56,178	Coniferous forest
8	36,938	56,094	Coniferous forest
9	37,044	56,277	birch forest
10	37,029	56,350	Mixed forest
11	37,176	56,306	Mixed forest
12	37,270	56,272	Mixed forest
13	37,213	56,110	suburban area.
14	37,340	56,025	Waste dump
15	37,299	55,914	Mixed forest

16	37,025	56,000	Mixed forest
17	36,780	55,988	Mixed forest
18	36,496	55,925	Mixed forest
19	36,439	56,112	Mixed forest
20	36,256	56,294	Mixed forest
21	36,061	56,204	Pine forest
22	35,853	56,267	Nature reserve Zavidovo, mixed forest
23	35,730	56,315	Birch forest
24	35,510	56,431	Birch an pine forest
25	35,621	56,279	Mixed forest
26	35,392	56,184	Mixed forest
27	35,643	56,074	Mixed forest
28	35,917	56,103	Mixed forest
29	36,322	56,029	Mixed forest
30	36,124	56,056	Mixed forest
31	35,810	56,027	Mixed forest
32	36,051	55,917	Mixed forest
33	35,797	55,819	Mixed forest
34	35,619	55,932	Mixed forest
35	35,501	56,022	Mixed forest
36	35,251	55,862	Mixed forest
37	35,541	55,759	Pine forest
38	35,318	55,564	Coniferous forest
39	35,431	55,417	Spruce forest
40	35,420	55,258	Spruce and birch forest
41	35,624	55,521	Spruce and birch forest
42	35,593	55,314	Spruce and birch forest
43	35,824	55,345	Spruce forest
44	35,809	55,641	Mixed forest
45	36,164	55,632	Spruce forest
46	36,252	55,714	Spruce forest
47	36,657	55,728	Mixed forest
48	36,856	55,729	Mixed forest
49	36,939	55,673	Mixed forest
50	36,764	55,601	Mixed forest
51	36,432	55,617	Mixed forest
52	36,339	55,624	Mixed forest
53	36,090	55,402	Spruce forest
54	36,000	55,212	Mixed forest
55	36,298	55,293	Mixed forest
56	36,664	55,387	Mixed forest
57	36,921	55,270	Mixed forest
58	36,942	55,454	Mixed forest
59	37,221	55,543	Spruce forest
60	37,405	55,541	Mixed forest
61	37,300	55,395	Mixed forest
62	37,159	55,245	Mixed forest
63	37,221	54,962	Mixed forest
64	37,828	54,875	Pine forest

65	37,668	54,901	Pine forest
66	37,468	54,965	Mixed forest
67	37,696	55,032	Deciduous forest
68	37,538	55,113	Coniferous forest
69	37,438	55,244	Coniferous forest
70	37,659	55,221	Deciduous forest
71	37,694	55,365	Coniferous forest
72	37,794	55,528	Coniferous forest
73	37,361	56,631	Mixed forest
74	37,427	56,784	Mixed forest
75	37,549	56,735	Coniferous forest
76	37,860	56,828	Spruce forest
77	37,823	56,735	Natural reservation "Zhuravlinaya rodina"
78	38,128	56,725	Mixed forest
79	38,236	56,630	Pine forest
80	38,099	56,459	Deciduous forest
81	37,870	56,543	Deciduous forest
82	37,645	56,501	Pine forest
83	37,628	56,391	Mixed forest
84	37,900	56,389	Mixed forest
85	38,347	56,454	Mixed forest
86	38,353	56,326	Mixed forest
87	38,105	56,371	Spruce forest
88	37,914	56,247	Spruce forest
89	38,216	56,218	Spruce forest
90	37,742	56,156	Mixed forest
91	37,805	56,118	Coniferous forest
92	38,082	56,129	Spruce forest
93	38,186	56,038	Pine forest
94	38,384	56,139	Coniferous forest
95	38,543	55,966	Mixed forest
96	38,481	55,883	Coniferous forest
97	38,242	55,827	Mixed forest
98	38,400	55,923	Mixed forest
99	38,464	55,711	Mixed forest
100	38,666	55,678	Pine forest
101	38,461	55,835	Spruce forest
102	38,695	55,871	Mixed forest
103	38,698	55,780	Mixed forest
104	38,922	55,890	Coniferous forest
105	39,036	55,830	Mixed forest
106	39,293	55,707	Pine forest
107	39,002	55,652	Deciduous forest
108	38,872	55,520	Pine forest
109	39,107	55,392	Spruce forest
110	39,144	55,532	Coniferous forest
111	39,410	55,596	Pine forest
112	39,714	55,725	Mixed forest
113	39,890	55,655	Pine forest

114	40,005	55,527	Mixed forest
115	39,669	55,566	Spruce forest
116	39,397	55,415	Coniferous forest
117	39,707	55,459	Birch forest
118	40,030	55,374	Pine forest
119	40,019	55,259	Mixed forest
120	39,735	55,186	Mixed forest
121	39,756	55,271	Mixed forest
122	39,601	55,352	Spruce forest
123	39,119	55,282	Spruce forest
124	39,434	55,238	Pine forest
125	39,605	55,137	Birch forest
126	39,383	55,035	Pine forest
127	39,161	54,899	Mixed forest
128	39,078	54,988	Pine forest
129	38,554	55,142	Mixed forest
130	38,267	55,110	Birch forest
131	38,421	55,239	Mixed forest
132	38,162	55,273	Mixed forest
133	38,094	55,441	Deciduous forest
134	37,947	55,573	Pine forest
135	37,951	55,361	Birch forest
136	37,990	55,159	Mixed forest
137	38,090	55,025	Mixed forest
138	38,322	54,871	Mixed forest
139	38,448	54,964	Birch forest
140	38,550	54,913	Mixed forest
141	38,739	54,951	Deciduous forest
142	38,834	55,180	Mixed forest
143	38,732	55,355	Mixed forest
144	38,502	55,444	Pine forest
145	38,655	55,538	Pine forest
146	38,370	55,624	Mixed forest
147	38,092	55,662	Mixed forest
148	38,020	55,862	Mixed forest
149	37,923	55,992	Pine forest
150	37,634	55,973	Spruce forest
151	37,580	56,080	Spruce forest
152	37,493	56,178	Deciduous forest
153	36,874	56,297	Mixed forest
154	37,657	56,312	Birch forest
155	37,595	56,653	Mixed forest
156	37,165	56,708	Spruce forest
